# Body Mass Index Trajectories among Middle-Aged and Elderly Canadians and Associated Health Outcomes

**DOI:** 10.1155/2016/7014857

**Published:** 2016-01-27

**Authors:** Meng Wang, Yanqing Yi, Barbara Roebothan, Jennifer Colbourne, Victor Maddalena, Peizhong Peter Wang, Guang Sun

**Affiliations:** ^1^Division of Community Health & Humanities, Faculty of Medicine, Memorial University, St. John's, NL, Canada A1B 3V6; ^2^Discipline of Medicine, Faculty of Medicine, Memorial University, St. John's, NL, Canada A1B 3V6

## Abstract

*Background*. Whether there is heterogeneity in the development of BMI from middle-age onward is still unknown. The primary aim of this study is to analyze long-term obesity and how BMI trajectories are associated with health outcomes in midlife.* Methods*. Latent Class Growth Modelling was used to capture the changes in BMI over time. In this study, 3070 individuals from the National Population Health Survey (NPHS), aged 40–55 years at baseline, were included.* Results*. Four BMI trajectory groups, “Normal-Stable” (N-S), “Overweight-Stable” (OV-S), “Obese I-Stable” (OB I-S), and “Obese II-Stable” (OB II-S), were identified. Men, persons of White ancestry, and individuals who had no postsecondary education had higher odds of being in the latter three groups. Moreover, members of the OV-S, OB I-S, and OB II-S groups experienced more asthma, arthritis, hypertension, diabetes, heart disease, cognitive impairment, and reduced self-rated overall health. Individuals in the OB II-S group were at greater risk for back problems, chronic bronchitis or emphysema, and emotional issues when compared to the N-S group.* Conclusion*. Understanding different BMI trajectories is important in order to identify people who are at the highest risk of developing comorbidities due to obesity and to establish programs to intervene appropriately.

## 1. Introduction

In 2011, Canadians aged 45 to 64 years had the highest self-reported rates of being overweight or obese, as high as 60% [1]. The annual cost of obesity-related diseases in Canada is substantial and estimated to be between $4.6 and $7.1 billion [2]. The impact of excess body weight on health gradually changes over one's lifetime [3]. Therefore, understanding how body weight changes over time, and the associated health conditions, is crucial for public health program development.

BMI trajectory analyses capture the changes of body weight over time. Changes in body weight can more accurately predict obesity-related health conditions when compared to static weight status [4, 5]. However, the majority of studies on the topic typically use Conventional Growth Modelling (CGM) to model BMI changes [6–10]. CGM uses one average pattern for the underlying population and estimates individual variability by random effects about the mean trend. In contrast to CGM, Latent Class Growth Modelling (LCGM) is a semiparametric statistical approach to quantify distinct BMI trajectories within a population [11, 12]. Previous studies have demonstrated the powerfulness and flexibility of LCGM for determining different BMI trajectory groups [13–16]. LCGM has been used to identify multiple BMI trajectories among children [13, 14] and adults [15, 16]. However, data regarding whether there is heterogeneity in BMI development from middle-age onward is still unknown.

There is growing interest in capturing different patterns of BMI changes over time. An important aim of this study is to test if BMI patterns carry differential morbidity potentials [16]. An awareness of distinct BMI trajectories allows identification of certain population groups as being at higher risk for obesity-related health conditions. This can then be used to target interventions to specific groups of people [17]. However, only two other studies have addressed the heterogeneity of BMI development based on LCGM and the potential associations with adverse health outcomes in midlife [15, 18]. These two studies reported that adverse health conditions were more prevalent in higher BMI trajectory groups; however, both studies were based on the US population. Østbye et al. identified four different BMI trajectories in adults aged 18–49 years. They investigated the associations between BMI trajectories and health outcomes when the respondents turned 40 years old [15]. Likewise, Finkelstein et al. identified four BMI trajectory groups for class I obese adults aged 25–33 years [18]. However, Finkelstein's study is not representative of the general population.

The primary aim of this study is to apply LCGM to analyze long-term BMI trajectories in an 18-year longitudinal study of middle-aged and older adults. This can then be used to examine the differential effects of BMI trajectories on health outcomes in midlife for the Canadian population.

## 2. Methods

The NPHS is a longitudinal health survey designed and implemented by Statistics Canada. The participants were household residents in the ten provinces of Canada and were followed up biennially from 1994 to 2011. Persons living on reserves and Crown Lands, residents of healthcare institutions, full-time members of the Canadian Forces Bases, and some of those who lived in remote areas of Ontario and Québec were excluded [19].

There were 3070 respondents, aged 40–55 years at baseline, used to identify the BMI trajectories for middle-aged to older adults. Respondents with at least four BMI records were included; 15.7% of participants were excluded. For the excluded group, the cumulative attrition rate to cycle four was 86.7%. For the included group, the cumulative attrition rate to cycle four was 13.2%. Therefore, respondents were excluded in this analysis mainly due to nonresponse to the NPHS. The respondents included were not significantly different from those excluded by the variables of age, gender, physical activity level at baseline, health conditions (e.g., asthma, arthritis or rheumatism (excluding fibromyalgia), back problems (excluding fibromyalgia and arthritis), high blood pressure, chronic bronchitis or emphysema, diabetes mellitus, and cognitive or emotional conditions), and overall self-rated health when they reached age of 55 years. On the other hand, differences were detected between the two groups in terms of ethnic/racial background, education level, mean BMI, family income, and smoking.

### 2.1. Time Variable: Age

An Accelerated Longitudinal Design (ALD) [20] was adopted to construct the patterns of BMI changes over a period of 31 years (age: 40–70 years), using age as time axis. Previous studies have evidenced the advantages of ALD in studying age-related developmental changes over time through linking multiple cohorts together [21]. The data should be less affected by dropout within an ALD, compared to a single-cohort design, since sample attrition tends to accumulate over time during data collection [20].

### 2.2. Trajectory Variable: BMI

Height and weight were self-reported in the NPHS and BMI was calculated as weight in kilograms divided by height in meters squared. The imputed BMI data provided by Statistics Canada was used in this analysis to define measures of BMI at each age. Detailed information on BMI imputation by the NPHS can be provided upon request [19].

### 2.3. Risk Factors

Gender (women or men), race/ethnicity (White or Aboriginal or other races), and education (education level less than high school, high school graduation, some college, or college and above) were included as risk factors. It is assumed that risk factors have a potential impact on group membership of trajectories. The preliminary analysis showed that BMI trajectory patterns did not change substantially by gender. Therefore, the BMI trajectories were modelled in the same model for both men and women and gender was considered as a risk factor of group membership.

Other risk factors include the probability of being physically active, smoking, and living with low income. These three latent variables were defined from the trajectories of the physical activity (PA) level, smoking, and income status variable in each cycle of the NPHS. During each cycle, the NPHS also collected information on the respondents' current smoking habits and total household income adjusted for the number of people living in the household. Detailed definitions of these variables can be provided upon request [19]. LCGM was used to identify different patterns of the probability of being physically active, smoking, and living with low income.

### 2.4. Health Outcomes

To test whether the prevalence of and risk of developing a health condition varied with different BMI trajectory groups, the self-reported health outcomes of individuals, at the age of 55 years in the NPHS, were used. The associations of health conditions and overall self-rated health with BMI trajectories were analyzed. The health outcomes included allergies, asthma, arthritis or rheumatism (excluding fibromyalgia), back problems (excluding fibromyalgia and arthritis), high blood pressure, migraine headaches, cancer, cataracts, chronic bronchitis or emphysema, diabetes mellitus, heart disease, and cognitive or emotional conditions. Emotional problems (self-perceived happiness) were assessed using the following categories: happy and interested in life, somewhat happy, somewhat unhappy, unhappy with little interest in life, and so unhappy that life is not worthwhile. Cognitive problems were ascertained by asking the questions “How would you describe your usual ability to remember things?” and “How would you describe your usual ability to think and solve day-to-day problems?” Six categories of self-reported cognitive status were ranked from no cognition problems to unable to remember or to think.

### 2.5. Statistical Analysis

LCGM was used to identify the most likely BMI trajectories and the three latent variables for the studied population [12]. All analyses were conducted using SAS version 9.3 (SAS Institute). The modelling selection process of BMI trajectories followed the criteria presented in the authors' previous work [22]. LCGM was also used to define three latent variables to identify different patterns of the probability of being physically active, smoking, and living with low income. A three-trajectory model for the probability of being physically active (active, moderately active, and inactive trajectories), smoking (smoking decreasing, smoking stable, and no smoking trajectories), and living with low income (high income and low income trajectories) was identified based on LCGM. Detailed model results of these latent variables are available upon request.

Each individual was assigned to a specific BMI trajectory group for which he or she was most likely to follow. Similar analysis was completed for the three latent variables. Multinomial logistic regression analyses were used to examine the impact of potential risk factors on the membership of the identified BMI trajectory groups while controlling for the other risk factors. Any potential interactions were tested for each pair of variables among the risk factors. Significant terms were retained in the model (*P* < 0.05). Furthermore, to examine whether the prevalence of and relative risk for an adverse health condition differed by BMI trajectory group, chi-square tests and bivariate regression analyses were conducted. The analyses were appropriately weighted using the survey sampling weights and bootstrap weights recommended by Statistics Canada.

#### 2.5.1. Ethical Considerations

This research was approved by Statistics Canada.

## 3. Results

Among the 3070 individuals included in this study, 48.5% were women. Most respondents self-identified as White (90.9%) while 0.44% were from an Aboriginal population. The majority (62.5%) of this population had some postsecondary education. From 1994 to 2011, the weighted prevalence of being overweight (BMI 25.0–29.9), obese class I (BMI 30.0–34.9), obese class II (BMI 35.0–39.9), and obese class III (BMI ≥ 40.0) increased from 41.8% to 42.2%, 12.2% to 18.9%, 2.8% to 5.8%, and 1.2% to 2.4%, respectively. On the other hand, the weighted prevalence of being underweight (BMI < 18.5) and normal weight (BMI 18.5–24.9) declined from 1.6% to 1.1% and 40.5% to 29.5%, respectively.

**Figure 1:**
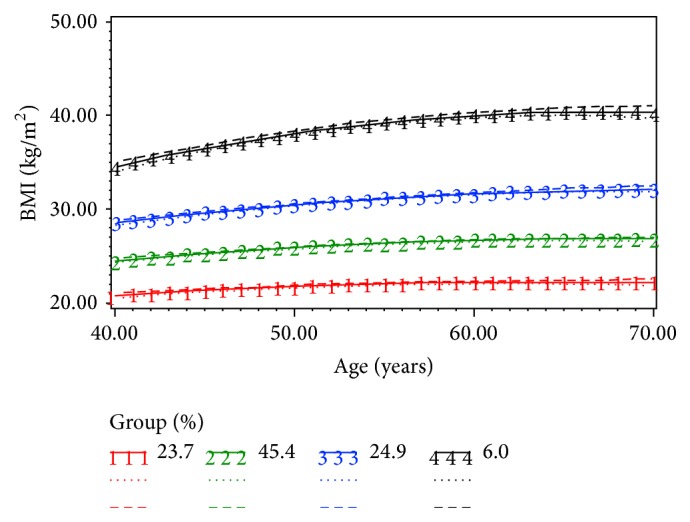
BMI trajectories for adults aged 40–70 years with 95% confidence intervals (four-group model, no covariates included) (NPHS, 1994–2011).

Four BMI trajectory groups, “Normal-Stable” (N-S), “Overweight-Stable” (OV-S), “Obese I-Stable” (OB I-S), and “Obese II-Stable” (OB II-S), were identified as most accurately characterizing the long-term patterns of BMI change. Trajectory results, including estimated parameters, GMP and AvePP, are shown in Table 1 and Figure 1. The AvePP of each trajectory group exceeds 0.95.

**Figure 2:**
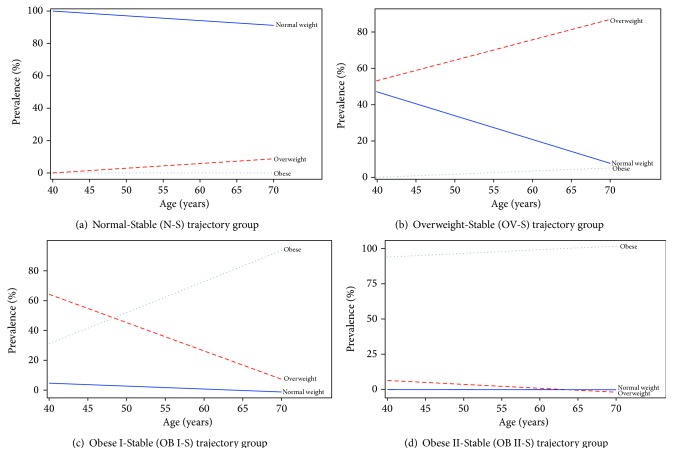
Weighted prevalence of body mass categories (under/normal weight, overweight, and obese—defined by BMI) from age 40 to 70 years, by body mass trajectory group, NPHS, 1994–2011.

The GMP of the N-S group was 23.7% (marked by number “1” in Figure 1). Most individuals assigned to this group remained underweight or of normal weight from age 40 to 70 years (Figure 2(a)).

The GMP of the OV-S group was 45.4% (marked by number “2” in Figure 1). This group started with an average BMI of 24.8 at 40 years of age and then increased and remained overweight thereafter. Within the OV-S group, the proportion of individuals who were overweight was greater than 50% at each age from 40 to 70 years. Approximately 30% of the individuals had a BMI of less than 25 until the age of 50 years. The proportion of obese individuals in this group did not exceed 10% at any age (Figure 2(b)).

The GMP of the OB I-S group was 24.9% (marked by number “3” in Figure 1). This group started with an average BMI of 27.9 (overweight) at age of 40 years and increased to an average BMI of 30.5 (obese class I) at age of 50 years. This group never reached obese class II status through ages 40–70 years. The proportion of underweight or normal weight individuals in this group was less than 20% at all ages. Additionally, 45% of the individuals in this group were overweight around the age of 40. This proportion declined to 30% by 60 years of age. Furthermore, the proportion of obese respondents in this group was about 50% by age 27 years and exceeded 70% by age 60 years (Figure 2(c)).

The GMP of the OB II-S group was 6.0% (marked by number “4” in Figure 1). This group started with an average BMI of 34.7 at 40 years of age and increased and remained in the obese class II category through ages 40–70 years. The weighted prevalence of underweight, normal weight, and overweight individuals was negligible for all ages in this group. Most respondents in this group were obese at the age of 40 years. The proportion of obese individuals in this group kept increasing through ages 40–70 years (Figure 2(d)).

After adjusting for multiple potential risk factors, other races (non-White or Aboriginal) (OR, 0.15; 95% CI, 0.04 to 0.58), some college (OR, 0.55; 95% CI, 0.42 to 0.73), college graduation and above (OR, 0.61; 95% CI, 0.47 to 0.80), the membership of the highest probability of being physically active (OR, 0.74; 95% CI, 0.57 to 0.95), and smoking (OR, 0.74; 95% CI, 0.57 to 0.95) were associated with a decreased risk of being in the Overweight-Stable (OV-S) trajectory group (Table 2). All of the above risk factors exerted similar effects on Obese I-Stable (OB I-S) and Obese II-Stable (OB I-S) groups, except for other races and education. Only the interaction term between gender and the probability of living with low income trajectory (*P* = 0.001) was found to be significant (data available upon request). Female gender was associated with a decreased risk of OV-S (OR, 0.29; 95% CI, 0.23 to 0.37), OB I-S (OR, 0.25; 95% CI, 0.19 to 0.32), and OB II-S (OR, 0.62; 95% CI, 0.40 to 0.95) among people who were assigned to the trajectory of high income; similar findings were found among members of the low income trajectory except for the OB II-S group.

The prevalence of adverse health conditions was the highest among the OB II-S group, followed by the OB I-S and OV-S groups, and the lowest among the N-S group. Members of the OV-S, OB I-S, and OB II-S groups were more likely than N-S group members to self-report asthma, arthritis, hypertension, diabetes, heart disease, cognitive issues, and reduced overall health. Furthermore, members of the OB II-S group were more likely to report chronic bronchitis or emphysema and emotional issues compared to members of the N-S group (Table 3). The prevalence of allergies, migraine headaches, cancer, or cataracts was not significantly different among the four BMI trajectory groups (data available upon request).

Compared to adults in the N-S group, those in the highest BMI trajectory group, OB II-S, were two times more likely to report asthma (OR = 2.6; 95% CI, 2.56–2.63), back problems (OR = 1.28; 95% CI, 1.26–1.29), chronic bronchitis or emphysema (OR = 2.52; 95% CI, 2.48–2.57), and cognitive limitations (OR = 1.58; 95% CI, 1.56–1.59). Additionally, individuals in the OB II-S group were nearly four times more likely to experience arthritis or rheumatism (OR = 3.79; 95% CI, 3.76–3.83) and heart disease (OR = 3.75; 95% CI, 3.69–3.82). Furthermore, those in the OB II-S group were eight times more likely to experience hypertension (OR=8.03; 95% CI, 7.95–8.11) and 26 times more likely to self-report diabetes (OR = 25.56; 95% CI, 25.10–26.03). Those in the OB II-S group were four times more likely to rate themselves as “unhappy” and two times more likely to rate themselves as “somewhat happy” compared to “happy” in the N-S group (OR = 3.90; 95% CI, 3.83–3.98 and OR = 1.88; 95% CI, 1.87–1.90, resp.). Moreover, those individuals in the OB II-S group were 9.53 times more likely to rate their overall health as “poor,” 11.48 times more likely to rate their overall health as “fair,” 5 times more likely to rate their overall health as “good,” and 2.27 times more likely to rate their overall health as “very good” compared to “excellent” in the N-S group (95% CI, 9.30–9.76; 95% CI, 11.27–11.69; 95% CI, 4.91–5.08; 95% CI, 2.24–2.31, resp.).

Overall, individuals in the OV-S, OB I-S, and OB II-S groups had higher risk for adverse health outcomes when compared to the N-S group. Some exceptions were that individuals in the OV-S group were 0.91 (95% CI, 0.90–0.91) times less likely to self-report back problems and that individuals in the OB I-S group were 0.38 (95% CI, 0.37–0.39) times less likely to report having chronic bronchitis or emphysema. Additionally, respondents in the OB I-S group were 0.90 (95% CI, 0.89–0.90) times less likely to rate themselves as “somewhat happy” compared to “happy” in the N-S group.

## 4. Discussion

There were four distinct BMI trajectory groups of middle-aged and older adults identified in this study. There was no significant weight gain or weight loss in the BMI trajectory groups. Findings of this study are in agreement with other BMI trajectory research based on LCGM [5, 16]. For instance, Zheng et al. identified six trajectory groups with a slight BMI increase over time for adults aged 51 to 77 years [5]. Similarly, Botoseneanu and Liang demonstrated that the change in BMI over time was moderate for people aged 51–61 years at baseline [16]. Further, people who were obese at the age of 40 were likely to keep excess weight during midlife without any interventions.

It was found that people who were not White or Aboriginal were less likely to follow higher BMI trajectory groups. This is in agreement with the documented evidence that the Aboriginal and White populations tend to have higher BMI measurements than other racial/ethnic groups in Canada [23]. By contrast, previous studies have reported that African Americans and Hispanics were more likely to follow higher BMI trajectories compared to the White population [15, 16, 18, 24]. This inconsistency may be due to the fact that these studies were based on the US population where there are cultural differences and larger populations of African Americans and Hispanics than in Canada [23]. In addition, respondents with some college education and college degrees were at a lower risk of being in the OV-S and OB I-S groups. This evidence is consistent with the study by Østbye et al. which found that a higher educational level lowered the BMI trajectory within each group [15]. The insignificance found in the highest BMI trajectory may be due to the small sample size of the OB II-S group.

The findings of this study contribute new evidence by using latent variables to capture the long-term impact of being physically active, smoking, and living with low income on BMI. People with the highest probability of being physically active during midlife were less likely to follow overweight and obese trajectories. This is consistent with the documented inverse relationship between body weight and physical activity [25]. The results show that smoking was associated with a decreased risk of gaining weight. Previous studies have determined that, on average, smokers weigh less than nonsmokers [26, 27]. Also, it was reported that smoking was associated with a lower BMI at baseline and a slower rate of change over time [28, 29]. Additionally, it was found that women were less likely to belong to higher BMI trajectory groups compared to men, though with one exception in the OB II-S group among people with low income; this finding is consistent with previous trajectory analyses [15, 16]. Conversely, some BMI trajectory studies reported no gender differences [29, 30]; however, these studies typically used one average BMI pattern to represent the whole population, which may conceal possible gender differences. The interaction between gender and income also confirmed the previous evidence that sociodeterminants of obesity differ by gender [31–34].

There is an increased risk for obesity-related health conditions among middle-aged and older adults with consistently high BMI measurements. It was found that people in the higher trajectory groups (i.e., OV-S, OB I-S, and OB II-S) were more likely to report asthma, arthritis, hypertension, diabetes, heart disease, cognitive conditions, and reduced overall health compared to their normal weight counterparts. However, only respondents in the highest BMI trajectory group (OB II-S) were at greater risk for back problems, chronic bronchitis or emphysema, and emotional issues. These findings are mostly consistent with previous evidence that demonstrated that people following a higher BMI trajectory were at a greater risk for various adverse health outcomes [15, 18, 24].

For example, Østbye et al. found that higher BMI trajectory groups had a higher prevalence of health conditions, including hypertension, diabetes, heart disease, arthritis, joint pain, asthma, back problems, and overall reduced self-rated health [15]. Finkelstein et al. also reported that obesity-related health conditions were more prevalent in higher BMI trajectory groups [18]. However, the prevalence of cognitive impairment, hypertension, and diabetes varied depending on the BMI trajectory group [24]. Clarke et al. demonstrated that respondents who were consistently overweight were at greater risk of being diagnosed with many chronic health conditions including hypertension, diabetes, asthma, lung disease, heart disease, and cancer [35]. Additionally, a systematic review showed that obesity was associated with developing asthma or worsening of asthma symptoms [36]. Crowson et al. concluded that obesity increased the odds of developing arthritis [37]; however neither García Rodríguez et al. nor Cerhan et al. found this association based on case control and cohort studies [38, 39]. These differing findings may be due to the different methods used by investigators and the fact that most prior studies did not consider the heterogeneity of BMI development.

The results suggest that respondents in the two obese BMI trajectory groups (OB I-S and OB II-S) were at greater risk for back problems; however, this was not the case for respondents in the overweight trajectory group (OV-S). Consequently, weight loss interventions aimed at treating or preventing back problems may need to be more strongly targeted towards obese individuals and overweight individuals, respectively. Furthermore, respondents in the higher BMI trajectory groups (OV-S, OB I-S, and OB II-S) had substantially higher risks for diabetes. This result is consistent with previous research findings that suggest that being overweight, even moderately, is an independent risk factor for developing numerous chronic diseases, especially diabetes [40]. Therefore, weight management to aid in the prevention of diabetes should target both overweight and obese individuals. Thus, an awareness of the different BMI trajectories is important in order to identify groups of people who are at the highest risk for certain conditions and diseases. Programs and interventions can then be targeted towards these groups to appropriately reduce morbidity and mortality.

This study has several limitations. Firstly, the use of self-reported height, weight, and health outcomes can introduce inaccuracies. Secondly, BMI can be an unreliable tool as a measure of a person's health or as an indication of what is considered a healthy body weight. Thirdly, the findings may underestimate the associations of excess weight and certain diseases since individuals under the age of 40 years were not included. Some studies have found that adolescent BMI may be a stronger predictor of future heart disease rather than adulthood BMI [40]. Additionally, results of this study cannot distinguish between type I and type II diabetes. In the NPHS, only the term diabetes was asked, but the reliability of self-reported diabetes mellitus has been previously validated [40, 41]. Lastly, the data were not adjusted to consider all confounders, such as genetic predisposition. Opportunities for future research can include using actual measures of excess body fat to determine the effects on the prevalence of certain diseases, studying the associations between childhood BMI and the development of certain health conditions in adulthood, and taking into consideration other factors of disease development such as genetics or lifestyle factors.

## 5. Conclusion

The findings suggest that being overweight or obese, especially over an extended period of time, adds risk for developing adverse health outcomes for the Canadian population. Individuals who are overweight or obese throughout their adulthood are at a greater risk for numerous health conditions compared with their healthy weight counterparts. Therefore, an awareness of different BMI trajectories is important in order to identify those people who are at the highest risk for certain diseases or conditions due to obesity. Interventions can then be effectively targeted to those people in order to reduce morbidity and mortality and to improve quality of life in adulthood.

## Figures and Tables

**Table 1:** Parameters estimated for BMI trajectories for adults aged 40–70 years (NPHS, 1994–2011).

The trajectory of BMI	Intercept-BMI at age 40 (s.e)	Linear term (s.e)	Quadratic term (s.e)	GMP	AvePP
N-S	21.51 (1.83)	0.31 (0.07)	−0.002 (0.0006)	23.7%	0.97
OV-S	24.76 (1.32)	0.42 (0.05)	−0.003 (0.0004)	45.4%	0.96
OB I-S	27.85 (1.83)	0.52 (0.07)	−0.004 (0.0006)	24.9%	0.96
OB II-S	34.73 (3.73)	1.09 (0.14)	−0.008 (0.001)	6.0%	0.98

**Table 2:** Odds ratios (OR) and confidence intervals (CI) for being categorized in the risk BMI trajectory groups (i.e., OV-S, OB I-S, and OB II-S) compared to N-S group by individual characteristics among adults in Canada, the NPHS, 1994–2011: result of multinomial logistic regression.

Risk factors	OV-S	OB I-S	OB II-S
	OR 95% CI	OR 95% CI	OR 95% CI
Age	0.99 (0.97–1.02)	0.96 (0.94–0.99)	0.99 (0.91–0.98)
Race/ethnicity			
Aboriginal (ref.)			
White	0.31 (0.09–1.14)	0.42 (0.10–1.76)	0.81 (0.08–8.10)
Other races	0.15 (0.04–0.58)	0.15 (0.03–0.65)	0.11 (0.01–1.46)
Education			
Less than high school (ref.)	1.0	1.0	1.0
High school	0.90 (0.64–1.25)	0.82 (0.58–1.19)	1.00 (0.57–1.76)
Some college	0.55 (0.42–0.73)	0.56 (0.41–0.77)	0.78 (0.48–1.25)
College and above	0.61 (0.47–0.80)	0.58 (0.43–0.78)	0.64 (0.40–1.03)
Physical activity (latent var.)			
Inactive (ref.)	1.0	1.0	1.0
Moderate active	0.91 (0.73–1.14)	0.90 (0.70–1.16)	0.50 (0.35–0.72)
Active	0.74 (0.57–0.95)	0.55 (0.41–0.74)	0.11 (0.06–0.20)
Smoking (latent var.)			
Nonsmoker (ref.)	1.0	1.0	1.0
Former smoker	1.20 (0.89–1.61)	1.05 (0.75–1.46)	1.19 (0.74–1.94)
Smoker	0.53 (0.42–0.66)	0.37 (0.28–0.49)	0.37 (0.28–0.58)
Living with low income			
Gender			
Men (ref.)	1.0	1.0	1.0
Women	0.48 (0.35–0.68)	0.65 (0.45–0.94)	1.21 (0.66–2.21)
Living with high income			
Gender			
Men (ref.)	1.0	1.0	1.0
Women	0.29 (0.23–0.37)	0.25 (0.19–0.32)	0.62 (0.40–0.95)

Relative adjusted odds ratios for membership in each trajectory using the N-S group as the reference class.

**Table 3:** Weighted prevalence and odds of self-reported health outcomes when respondents turned 55 years, by BMI trajectory group (NPHS, 1994–2011).

Health outcome	The trajectory of BMI								*P*-trend
	N-S (*N* = 728)		OV-S (*N* = 1394)		OB I-S (*N* = 764)		OB II-S (*N* = 184)		
	%a	ORb	%	OR (95% CI)	%	OR (95% CI)	%	OR (95% CI)	<.0001
Asthma^*∗∗*^	4.4	Ref.	7.1	1.65 (1.63–1.67)	9.2	2.18 (2.16–2.21)	10.8	2.60 (2.56–2.63)	<.0001
Arthritis or rheumatism^*∗∗*^	20.9	Ref.	26.9	1.39 (1.39–1.40)	30.3	1.65 (1.64–1.66)	50.0	3.79 (3.76–3.83)	<.0001
Back problems^*∗*^	19.6	Ref.	18.1	0.91 (0.90–0.91)	23.9	1.29 (1.28–1.30)	23.7	1.28 (1.26–1.29)	<.0001
Hypertension^*∗∗*^	12.3	Ref.	22.1	2.03 (2.02–2.04)	28.5	2.86 (2.84–2.88)	52.9	8.03 (7.95–8.11)	<.0001
Chronic bronchitis or emphysema^*∗∗*^	2.8	Ref.	3.1	1.12 (1.10–1.14)	1.1	0.38 (0.37–0.39)	6.7	2.52 (2.48–2.57)	<.0001
Diabetes^*∗∗*^	1.4	Ref.	5.7	4.30 (4.22–4.37)	9.6	7.49 (7.36–7.62)	26.6	25.56 (25.10–26.03)	<.0001
Heart disease^*∗∗*^	2.5	Ref.	5.5	2.29 (2.26–2.32)	6.0	2.49 (2.45–2.52)	8.7	3.75 (3.69–3.82)	<.0001
Cognitive issue^*∗∗*^	20.5	Ref.	21.5	1.06 (1.05–1.07)	24.7	1.27 (1.26–1.28)	28.9	1.58 (1.56–1.59)	<.0001
Emotional issue^*∗∗*^									
** **Happy	79.7	Ref.	77.5	Ref.	80.4	Ref.	64.9	Ref.	<.0001
** **Somewhat happy	17.8	Ref.	19.2	1.12 (1.11–1.12)	16.0	0.90 (0.89–0.90)	27.2	1.88 (1.87–1.90)	
** **Unhappy	2.5	Ref.	3.2	1.33 (1.31–1.35)	3.6	1.43 (1.40–1.45)	7.9	3.90 (3.83–3.98)	
Self-rated health^*∗∗*^									
** **Excellent	23.0	Ref.	18.8	Ref.	16.2	Ref.	6.4	Ref.	<.0001
** **Very good	38.8	Ref.	37.3	1.18 (1.17–1.18)	38.6	1.41 (1.40–1.42)	24.7	2.27 (2.24–2.31)	
** **Good	28.7	Ref.	30.7	1.31 (1.30–1.32)	32.5	1.61 (1.59–1.62)	40.1	5.00 (4.91–5.08)	
** **Fair	6.9	Ref.	8.5	1.49 (1.48–1.51)	10.3	2.11 (2.08–2.13)	22.3	11.48 (11.27–11.69)	
** **Poor	2.4	Ref.	4.6	2.33 (2.30–2.36)	2.3	1.35 (1.32–1.37)	6.5	9.53 (9.30–9.76)	

^*∗*^ *P* value < .10, ^*∗∗*^ *P* value < .01.

## Abbreviations

BMI: Body Mass Index
LCGM: Latent Class Growth Modelling
CGM: Conventional Growth Modelling
NPHS: National Population Health Survey
GMP: Group Membership Probability
AvePP: Average posterior probability of group membership
OR: Odds ratio
CI: Confidence interval.
